# Vitamin A in resistance to and recovery from infection: relevance to SARS-CoV2

**DOI:** 10.1017/S0007114521000246

**Published:** 2021-01-20

**Authors:** C. B. Stephensen, G. Lietz

**Affiliations:** 1 Immunity and Disease Prevention Research Unit, USDA Western Human Nutrition Research Center, and Nutrition Department, University of California, Davis, CA, USA; 2 Human Nutrition Research Centre, Population Health Sciences Institute, Newcastle University, Newcastle upon Tyne NE2 4HH, UK

**Keywords:** SARS-CoV2, COVID-19, Vitamin A, Retinoic acid, Immunity, Angiotensin-converting enzyme 2 (ACE2), Lipofibroblasts

## Abstract

SARS-CoV2 infects respiratory epithelial cells via its cellular receptor angiotensin-converting enzyme 2, causing a viral pneumonia with pronounced inflammation resulting in significant damage to the lungs and other organ systems, including the kidneys, though symptoms and disease severity are quite variable depending on the intensity of exposure and presence of underlying conditions that may affect the immune response. The resulting disease, coronavirus disease 2019 (COVID-19), can cause multi-organ system dysfunction in patients requiring hospitalisation and intensive care treatment. Serious infections like COVID-19 often negatively affect nutritional status, and the resulting nutritional deficiencies may increase disease severity and impair recovery. One example is the viral infection measles, where associated vitamin A (VA) deficiency increases disease severity and appropriately timed supplementation during recovery reduces mortality and hastens recovery. VA may play a similar role in COVID-19. First, VA is important in maintaining innate and adaptive immunity to promote clearance of a primary infection as well as minimise risks from secondary infections. Second, VA plays a unique role in the respiratory tract, minimising damaging inflammation, supporting repair of respiratory epithelium and preventing fibrosis. Third, VA deficiency may develop during COVID-19 due to specific effects on lung and liver stores caused by inflammation and impaired kidney function, suggesting that supplements may be needed to restore adequate status. Fourth, VA supplementation may counteract adverse effects of SARS-CoV2 on the angiotensin system as well as minimises adverse effects of some COVID-19 therapies. Evaluating interactions of SARS-CoV2 infection with VA metabolism may thus provide improved COVID-19 therapy.

## Graphical abstract

**Figure:**
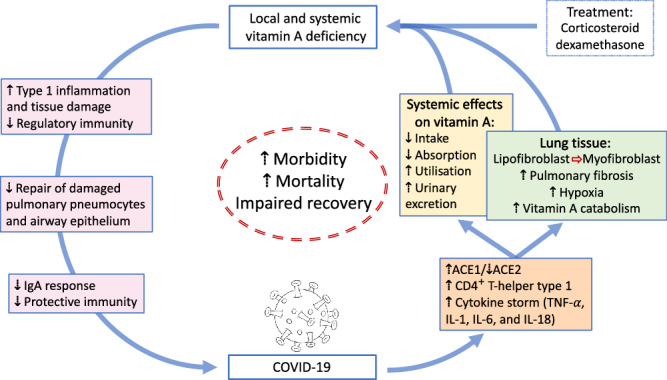

The Coronaviridae family of single-stranded RNA viruses includes four genera: *alpha*-, *beta*-, *gamma*- and *delta-coronavirus* ^(1)^. Seven coronaviruses are known to infect humans. Two (229E and NL63) from the alpha genus and two (OC43 and HKU-1) from the beta genus cause mild upper respiratory tract infections and symptoms of the common cold. The remaining three human coronaviruses are also in the beta genus but cause severe lower respiratory tract infections, respiratory failure and death. These viruses are MERS-CoV, which causes the Middle East respiratory syndrome; SARS-CoV1, which causes severe acute respiratory syndrome, and SARS-CoV2, which is causing the current pandemic of coronavirus disease 2019 (COVID-19)^(1,2)^. SARS-CoV2 spreads readily from person-to-person due to the long incubation period of 2–14 d following infection^(3)^ and, according to data available to the WHO, has infected 75 million people and caused 1·7 million deaths worldwide between December 2019 and December 2020^(4)^.

SARS-CoV2 infection causes both mild (in approximately 80 % of cases) and severe disease. Symptoms during mild disease include fever, dry cough, dyspnoea, sore throat, malaise, myalgia and, less frequently, gastrointestinal symptoms such as anorexia, nausea and diarrhoea. Mild cases are typically treated at home with supportive care, but no specific anti-viral therapy is currently recommended^(5)^. Some patients (approximately 20 % of all infections) progress from mild to more severe disease, the diagnosis of which involves a positive test for SARS-CoV2, tachypnoea and hypoxaemia and specific findings of decreased arterial oxygen concentration or a chest X-ray showing pneumonia, indicating extensive lung inflammation. Some patients with severe infection (approximately 5 % of all infections) develop renal failure and intravascular coagulation, require prolonged mechanical ventilation and may have involvement of multiple organ system failure. The survival rate in this most severe group is approximately 50 %^(6)^. Thus, SARS-CoV2 infection causes a multifaceted pathology, including heart attacks, coronary-related kidney damage and severe stroke in people with no previous history of CVD^(3)^. The most common complication of SARS-CoV2 is progressive consolidation of the lung leading to severe acute respiratory syndrome (SARS) due to extensive pulmonary fibrosis promoted by enhanced lipofibroblast–myofibroblast transition^(7)^.

Many therapeutic interventions are being evaluated in severely affected patients including anti-viral, anti-malaria and anti-inflammatory interventions. The anti-viral drug remdesivir reduces time to hospital discharge in severe affected SARS-CoV2 patients, but fails to reduce respiratory tract viral load^(8)^. On the other hand, the anti-viral drug umifenovir reduces virus entry into target cells by inhibiting fusion of viral envelope^(3)^. The anti-malaria drug hydroxychloroquine sulphate could also block SARS-CoV2 infection by preventing the fusion of the virus with the cell membrane^(3)^, but has shown to be associated with heart arrythmias^(9)^ and induce retinopathy in some patients, potentially through inactivating lysosomal enzymes, blocking the digestion ability of phagocytes, binding to melanin and blocking organic anion transporter family member 1A2 OATP1A2^(10)^. The anti-inflammatory corticosteroid dexamethasone was shown to reduce the death of SARS-CoV2-ventilated patients in the RECOVERY study by one-third^(3)^, but not in patients experiencing a milder disease^(8)^.

The purpose of this review is to examine how vitamin A (VA) status might affect host resistance to, or recovery from, COVID-19 disease. VA is of particular interest in this regard for at least three reasons. First, VA deficiency impairs host resistance to infection. VA was termed ‘the anti-infective’ vitamin approximately 90 years ago because of the increased risk of infections, particularly of lung infections, in VA-deficient experimental animals^(11)^, indicating that some aspect of host resistance to, or recovery from, infection was impaired. Research in the past 30 years has demonstrated that VA (retinol) is converted to its active metabolite retinoic acid by cells of the immune system and that retinoic acid then acts as an autocrine or paracrine factor to regulate many aspects of immune function, as has been reviewed recently^(12,13)^. Second, the action of VA is crucial in normal lung development and in repair of lung tissue subsequent to injury^(14)^; thus, VA status may be particularly important during recovery from COVID-19. Third, severe infections are known to have a significant, negative impact on VA status by several mechanisms, including reduced absorption^(15)^, decreased hepatic mobilisation^(16)^ and urinary loss of VA due to glomerular and/or tubular dysfunction^(17)^. Since severe COVID-19 cases often have prolonged hospitalisation with renal involvement, it is plausible that VA deficiency could develop during this period of severe disease. If this is the case, and VA deficiency impairs host resistance to infection and recovery of normal lung function, then recovery from severe COVID-19 disease might be improved by nutritional support involving VA supplementation, as well as other appropriate nutritional support. Fourth, medication used to reduce the impact of severe COVID-19 infection could alter the metabolism of VA in affected tissues and therefore induce tissue-specific VA deficiency^(18)^, which could reduce the ability to repair affected tissues^(14)^.

## SARS-CoV2 pathogenesis and effects of vitamin A on the immune system

### SARS-CoV2 infection of the respiratory epithelium

Similarly to SARS-CoV1, the cellular infection mechanism of SARS-CoV2 is mainly mediated by the cell-surface receptor angiotensin-converting enzyme 2 (ACE2)^(19)^. SARS-CoV2 uses ACE2 for receptor-mediated cell entry and serine protease transmembrane serine protease 2 (TMPRSS2) for S protein priming^(20)^. Cell entry of SARS-CoV2 can be partially blocked by the clinically proven serine protease inhibitor camostat mesylate, which is active against serine protease transmembrane serine protease 2 (TMPRSS2)^(20)^. Although very low levels of ACE2 protein have been observed in the normal respiratory system^(21)^, the presence of ACE2 protein levels and viral RNA has been confirmed in ciliated epithelial cells, indicating that low-level protein expression in upper airway epithelial cells facilitates infection of ciliated cells, followed by a rapid interferon-induced increase of ACE2 expression in lower airway ciliated and type-2 alveolar epithelial cells, potentially allowing SARS-CoV2 to spread across the respiratory mucosa^(22)^. The cell-surface ACE2 receptor internalises on binding to the SARS-CoV spike protein, leading to ACE2 receptor down-regulation^(23,24)^. The SARS-CoV spike protein-mediated ACE2 down-regulation could contribute to the severity of lung pathologies such as pulmonary fibrosis and acute respiratory distress syndrome, since the counterbalance of ACE2 on angiotensin II production in the renin–angiotensin system is deregulated, affecting its ability to modulate the innate immune system and to regulate inflammation^(19,25,26)^. ACE2 knockout mice infected with SARS-CoV1 show reduced pathological alterations in the lung^(19)^. Increased incidence of intravascular coagulation with SARS-CoV2 infection could also be mediated via ACE2, since ACE2 deficiency is associated with up-regulation of putative mediators of atherogenesis, such as cytokines and adhesion molecules, and reduced ACE expression is observed in established atherosclerotic plaques^(27)^. Finally, the observed difference in SARS-CoV2 severity between children and adults could in part be attributed to the lower expression of the SARS-CoV2-targeted receptor ACE2 and serine protease serine protease transmembrane serine protease 2 (TMPRSS2) in nasal and bronchial airways in children compared with adults^(28)^.

### Vitamin A and epithelial barriers

VA is needed to maintain healthy respiratory and intestinal epithelial barriers^(17)^. VA deficiency may not produce obvious changes in healthy epithelial surfaces, but following viral infection in the intestine, for example, pathological changes to infected epithelial surfaces were found to be much greater in VA-deficient than in control mice^(29)^. In the respiratory tract, VA deficiency also increases epithelial damage and impairs recovery, sometimes leading to squamous metaplasia in alveoli and airways, following noxious exposures such as ozone treatment^(30)^ and infection with influenza virus^(31)^. Sputum (mucus) also provides a solid physical barrier to pathogens^(32)^ and contains many of the macromolecules of the innate immune system^(33)^. Sputum is a mixture of oligomeric mucins, MUC2, MUC5AC and MUC5B which are synthesised and secreted by the differentiated mucociliary epithelium^(34)^. Regulation of mucin production is induced by all-*trans* retinoic acid via retinoic acid receptor alpha as the major retinoid receptor subtype^(35)^. Importantly, moderate VA supplementation, but not high-dose VA supplementation or VA deficiency, improves mucin secretion by regulating the gene expression of cytokines and epithelial growth factors^(36)^.

The VA metabolite all-*trans* retinoic acid has also been shown to up-regulate the expression of ACE2^(37,38)^, resulting in the reduction of blood pressure and the attenuation of myocardial damage in spontaneously hypertensive rats^(37)^. Increased levels of ACE2 after VA supplementation could have two effects: 1) it could increase the risk of SARS-CoV2 infection at the time of virus exposure^(39)^, particularly in individuals who have an adequate VA status; or 2) it could reduce the risk of sympathetic over-activation seen in severe SARS-CoV2 infection, obese and diabetic patients^(40–42)^ through VA supplementation that activates the ACE2-Ang 1–7-Mas axis.

VA is also important during embryonic lung development^(43)^; thus, the role in tissue repair in postnatal life is not surprising. A recent review highlights the potential role of VA and retinoic acid as possible therapeutic interventions to prevent pulmonary fibrosis following lung injury^(44)^, suggesting a role for VA in recovery from severe COVID-19 disease. Lipofibroblasts are the retinoid storage cells of the lung, similar to stellate cells of the liver^(18)^, and contain many components of the retinoid signalling pathway including receptors and binding proteins^(45)^. Importantly, the defining characteristic of hepatic fibrosis is the loss of VA from stellate cells^(46)^. Similarly, chronic nutritional VA deficiency results in pulmonary fibrosis through decreased alveolar septation, squamous metaplasia of the respiratory epithelium and a thickening of the alveolar basement membrane followed by an increase in collagens and a deposition of ectopic collagen fibrils^(14)^. Pulmonary fibrosis is characterised by conversion of lipofibroblasts to FGF10-expressing myofibroblasts localised to *α*SMA expressing cells, down-regulation of PPAR*γ* and activation of TGF-*β* signalling^(47)^. PPAR*γ* activation reverts fibrosis in both the lung and the liver through inhibition of *α*SMA, type I collagen and TGF-*β* expression^(46,48)^. The beneficial effects of retinoids on pulmonary fibrosis may be through activation of the retinoid X receptor via 9-*cis*-retinoic acid^(49)^.

### Innate immune response to SARS-CoV2 infection

The immune response to respiratory viral infections, including coronaviruses, is initiated by barrier defences at the site of initial virus replication^(50)^. SARS-CoV2 infects ciliated epithelial cells in the upper respiratory tract and alveolar type II pneumocytes in the lower respiratory tract via its cellular receptor ACE2^(51)^. SARS-CoV2 thus first engages the innate immune system in respiratory epithelial cells and adjacent macrophages. Viral RNA in the cytoplasm or endosomes is recognised by pattern-recognition receptors including RIG-1, TLR3, TLR7 and TLR9. These receptors trigger transcription of type I and type III interferon (IFN) which act via cellular receptors to induce anti-viral programmes in infected and adjacent cells that render these cells refractory to viral infection. However, coronaviruses, including SARS-CoV2, can act on these pathways to block induction of anti-viral activity, which may be a factor in the development of more severe diseases. In addition to type I and III IFN, pattern-recognition receptor activation triggers production of other cytokines, including TNF-*α*, IL-1, IL-6 and IL-18 by innate immune cells, particularly macrophages. These cytokines help initiate inflammation, including recruitment of other immune cells to the site of initial infection. Innate lymphoid cells found at tissue sites are often involved in the initial response to viral (and other) infections, but their role in SARS-CoV2 infection is uncertain^(52,53)^.

### Effects of vitamin A on innate immunity

Many cells of the innate immune system, including innate lymphoid cells, macrophages and granulocytes, are affected by VA. Type 1 innate lymphoid cells, including natural killer (NK) cells, play a role in responses to viral infection and their activity and number in peripheral blood is decreased by VA deficiency^(54)^, while retinoic acid treatment can also dampen activity^(55)^. Neutrophil and macrophage function is also directly affected by VA. Phagocytic and bacterial-killing activity of these cells is impaired by VA deficiency, leading to decreased resistance to some infections, though production of the CD4^+^ T-helper type 1 (Th1)-promoting cytokine IL-12 can be increased, causing increased type 1 inflammation^(17)^. *Ex vivo* and animal studies show that treatment of macrophages with retinoic acid can have anti-inflammatory effects, dampening production of IL-12 and pro-inflammatory cytokines such as TNF-*α*, as well as increasing production of the regulatory cytokine IL-10^(55)^. Other effects on macrophage phenotype are also caused by retinoic acid. For example, in *Leishmania* infection of mice, retinoic acid treatment diminishes the development of M1 macrophages, a component of protective type 1 immunity in this model system, and increases the development of M2 macrophages and type 2 immunity, which is not protective. Thus, retinoic acid intervention in this system decreases resistance to infection^(56)^. Conversely, in *Schistosoma mansoni* infection of mice, VA deficiency exacerbates disease due to excessive inflammation and tissue damage, while retinoic acid treatment increases conversion of macrophages into a tissue-resident phenotype that dampens inflammation and allows control of infection and enhancement of tissue repair^(57)^. While many of the effects of retinoic acid on innate immune cells can be seen as anti-inflammatory, a lesson from these and other studies is that intervention with retinoic acid during infections can have unpredictable effects on the course of a particular infection depending on the specifics of the immune response that are responsible for pathogen clearance. This topic has been discussed in a recent review^(58)^.

### Adaptive immune response to SARS-CoV2 infection

Dendritic cells link the initial anti-viral response of the innate immune system to development of adaptive immunity, involving B and T lymphocytes, that will help clear infections that cannot be resolved by innate immunity alone. Adaptive immunity also has a memory component that, in the case of most acute viral infections, provides a rapid and robust response upon subsequent exposure to the same virus, typically providing protection against symptomatic infection. Such responses develop for other coronaviruses^(50)^, and hopefully, this will also be true of SARS-CoV2.

To initiate adaptive immunity, dendritic cells at the site of infection are also activated by pattern-recognition receptor-mediated recognition of viruses, as well as by local cytokine production, and deliver viral antigen from the site of infection to a regional lymph node to initiate development of virus-specific effector and memory T cells, as well as memory B cells that results in development of antibody-producing plasma cells. Immunologists recognise three basic types of protective immunity against infectious diseases, types 1, 2 and 3^(59)^. Type 1 immunity is typically induced in response to viruses and other intracellular pathogens and involves development of Th1 cells producing the signature cytokine IFN-*γ* and CD8^+^ cytotoxic T-lymphocyte which can directly kill virus-infected cells, and a robust serum IgG antibody response, often targeting the epitope of the viral glycoprotein involved in binding to its cellular receptor (the spike [S] protein in the case of SARS-CoV2), thus neutralising the ability of the virus to infect cells. Macrophages activated by Th1 cells at sites of infection are also a key component of type 1 immunity. Type 2 immunity provides protection against intestinal parasites, involves development of CD4^+^ Th2 cells producing IL-4, -5 and -13, induction of an antibody response involving IgE and activation of eosinophils and basophils at mucosal surfaces, near the sites of pathogen exposure. Type 3 immunity targets extracellular pathogens, involves development of CD4^+^ Th17 cells producing IL-17 and IL-22, development of appropriate antibody responses and engagement of mucosal epithelial cells and neutrophils to provide barrier protection. CD4^+^ follicular-helper T cells develop during all of these responses to provide help for development of antigen-specific memory B cells and a robust antibody response. CD4^+^ regulatory (Treg) cells also develop during most adaptive responses to provide a pathogen-specific cell type capable of dampening pro-inflammatory immunity by direct cell–cell interaction or production of the cytokines IL-10 and TGF-*β*.

Research on the adaptive immune response to SARS-CoV2 is in an early state, but two recent reviews indicate that patients recovering from COVID-19 have the Th1, CD8^+^ cytotoxic T-lymphocyte and neutralising antibody responses expected of a typical type 1 response against a viral infection^(52,60)^. Briefly, Th1 responses predominate over Th2 and Th17, as expected, and viral epitopes from all major structural proteins, including spike (S), matrix (M) and nucleocapsid (N), are seen in recovering patients. CD8 T-cell responses are also seen against the same proteins. The magnitude of the response is somewhat greater in patients with more severe disease, which could be due to greater antigenic stimulation. Similar patterns of response were seen against SARS-CoV1 in recovered SARS patients^(61)^, and protection against death by CD8 cells was demonstrated in a mouse model^(62)^. Similar CD4 and CD8 responses were seen against MERS-CoV in MERS survivors 0·5–2 years after infection^(63)^. These findings suggest that patients recovering from SARS-CoV2 infection may have protective T-cell memory responses. In contrast to the potential benefit of such Th1 responses, some evidence from SARS patients^(64)^ as well as preliminary work from COVID-19 patients cited in a recent review^(60)^ suggests that Th2 responses may be associated with poor outcomes to infection. B-cell responses develop in response to SARS-CoV2, as demonstrated by the serum IgM and IgG antibody responses that are seen within 2 weeks of infection, as is also seen with MERS-CoV and SARS-CoV1^(65)^. Antibodies are directed primarily against the S and N proteins, and the ACE2-binding site is quite immunogenic, resulting in robust neutralising antibody responses^(52)^.

### Effects of vitamin A on adaptive immunity

As discussed above, dendritic cells are a key bridge from innate to adaptive immunity during infection and immunisation. Dendritic cells provide the signals necessary to stimulate development of pathogen-appropriate adaptive immune responses. These signals include peptide antigen to provide pathogen-specificity (i.e. recognition of a specific strain of the flu virus by memory T cells), co-stimulation to insure continued proliferation of developing T cells and specific cytokines to steer CD4 T-cell development towards the pathogen-appropriate Th1, Th2, Th17, Treg and CD4^+^ follicular-helper T phenotypes. In addition, a fourth signal that is often provided by dendritic cells, particularly in the intestinal and other mucosal-associated immune tissues, is retinoic acid^(66)^, the production of which is diminished during VA deficiency. Retinoic acid affects several aspects of T-cell development, including modification of T-cell phenotype development and mucosal homing of lymphocytes. These effects have recently been reviewed^(12)^. They are pleiotropic and depend on modifying factors (e.g. the local cytokine environment), but several main points are clear.

Perhaps, the most consistent and biologically important effect of retinoic acid produced by dendritic cells, and a principal reason for retinoic acid production being constitutive in mucosal dendritic cells, is that retinoic acid induces transcription of two mucosal-homing molecules on lymphocytes, chemokine receptor 9 and *α*4*β*7 integrin. Thus, memory T cells, B cells and antibody-producing plasma cells which develop in the intestine or respiratory tract (both are initiating sites of the common mucosal immune response) will preferentially recirculate to these sites, whereas T cells developing from systemic lymph nodes are less likely to do so^(66)^. Mucosal infections often trigger secretory IgA responses, and the plasma cells responsible for secreting IgA are found at submucosal sites due to the imprinting of chemokine receptor 9 and *α*4*β*7 integrin expression. This mechanism may explain why VA deficiency sharply reduces the secretory IgA response to influenza A virus infection in the nasal mucosa of mice^(67)^. Furthermore, impaired IgA antibody and infrequent virus-specific CD8^+^ T response following respiratory virus infection induced a cytokine storm in the upper respiratory tract of VA-deficient mice^(68)^. Retinoic acid also enhances intestinal homing of other cell types, including Treg cells^(69)^.

Second, VA deficiency has contrasting effects on Th1/Th2 development, with deficiency not affecting or sometimes enhancing Th1 development (via increased IL-12 production, as discussed above) but dampening Th2 development, particularly during the early stages of phenotype commitment immediately after antigenic stimulation. In contrast, retinoic acid, or high-level dietary VA, can enhance type 2 immunity^(70)^. However, retinoic acid, at a somewhat later stage in development of Th1 memory cells, can help stabilise phenotype commitment to the Th1 lineage^(71)^. Similarly, effects of VA on CD8^+^ cytotoxic T-lymphocyte development, a key component of type 1, anti-viral immunity are mixed^(12)^. Using model viral infections to explore the effects of VA deficiency has shown no impairment of clearance of acute influenza A virus infection in mice, a slight enhancement of some aspects of the type 1 response squamous metaplasia during recovery^(31)^. Using chronic lymphocytic choriomeningitis virus infection in mice also showed an elevated type 1 response, more severe pathological changes, higher virus load and increased mortality, effects which were reversed with retinoic acid^(72)^.

Third, VA regulates the balance between Th17 and Treg development. Both cell types often develop in the intestinal lymphoid tissue (as well as at other sites) with Treg cells being predominant when inflammation is minimal and constitutive TGF-*β* and retinoic acid production bias memory T-cell development towards a Treg phenotype which is appropriated for antigens derived from food and commensal bacteria. However, when inflammation develops, IL-6 is produced and this shifts the balance towards Th17. During VA deficiency, Treg development is diminished because retinoic acid production is reduced^(55)^.

Fourth, retinoic acid affects B cell development, and VA deficiency decreases the antibody response to T-cell-dependent antigens, in particular^(12)^. While VA deficiency in mice slightly enhances type 1-related IgG2a antibody responses, the IgG1 response is diminished and, in particular, the IgA response is specifically diminished because retinoic acid can enhance class-switching to IgA^(73,74)^.

### Contribution of the immune system to COVID-19 pathogenesis

Approximately 5 % of COVID-19 patients, many with underlying conditions such as CVD, diabetes mellitus and obesity, develop severe disease with a high risk of death. Male sex and age above 65 years are also risk factors^(6)^. The pathogenesis of severe disease involves excessive activation of the immune system that can result in immunopathology that increases disease severity, as has been reviewed recently^(52,75–77)^. During the initial innate immune response, a robust IFN type I/III response appears to predict a milder course of disease, presumably by controlling initial virus replication and allowing development of the adaptive response. However, patients with severe disease are more likely to have a lower initial IFN type I/II response (though it may be elevated later) which may result in a failure to control initial viral replication. Prolonged viral replication then appears to result in high-level production of pro-inflammatory cytokines (including TNF-*α*, IL-1, IL-6 and many others), likely produced by activated macrophages but perhaps by other innate cells such as neutrophils that may be attracted to the lungs. This local hyper-inflammation may dampen development of adaptive immunity and in patients with severe disease lymphopenia, including low levels of T cells, is pronounced. During this ‘cytokine storm’ with activation of innate immune cells, a system response develops that includes elevation of acute phase proteins, particularly CRP, and activation of the complement system which may result in vascular leakage as well as diffuse intravascular coagulation which can cause organ damage thus increasing disease severity. Developing therapeutic interventions to dampen this immune-mediated pathology is a high priority of current research. Potential drug targets include the NF-κB pathway to attenuate TNF-*α* and IL-6 expression, the JAK/STAT signalling pathway and the sphingosine-1-phosphate receptor 1 pathway^(78)^. The JAK/STAT and sphingosine-1-phosphate receptor 1 signalling pathways are of particular interest since SARS-CoV2 potential down-regulation of ACE2 expression could result in over-production of angiotensin II via the related ACE enzyme, leading to enhanced IL-6 production. JAK/STAT inhibitors such as Baricitinib or sphingosine-1-phosphate receptor 1 receptor agonists such as Fingolimod could attenuate the cytokine storm^(78)^.

### Possible implications of vitamin A deficiency for immune response to SARS-CoV2

The effects of VA in the immune system discussed above have several possible implications for individuals with VA deficiency infected by SARS-CoV2. First, VA deficiency is not likely to impair a type 1 anti-viral response to SARS-CoV2, but could increase the severity of type 1 inflammation and tissue damage in the lungs following viral infection. Second, after clearance of SARS-CoV2 infection, VA deficiency could impair repair of damaged alveolar pneumocytes and airway epithelium. Third, protective immunity to SARS-CoV2 could be impaired by VA deficiency, particularly the mucosal IgA response, which could be important in resistance to reinfection, though development of memory Th1 and CD8^+^ cytotoxic T-lymphocyte response might also be affected.

## Effect of infections on vitamin A metabolism and status

**Fig. 1.**
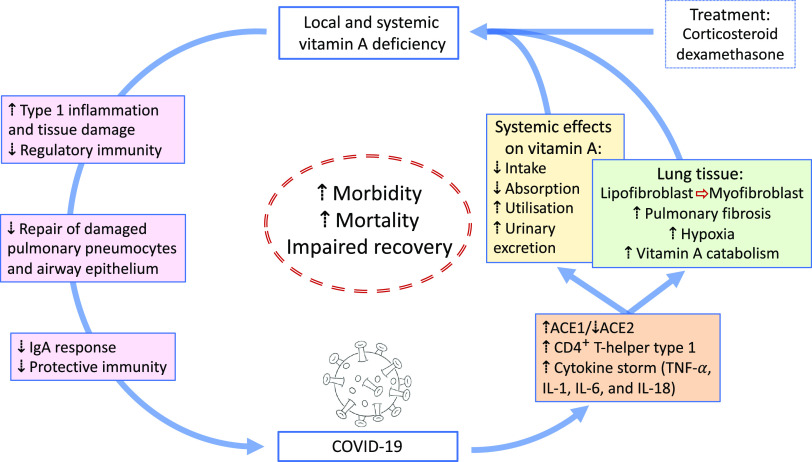
Overview of potential interactions between vitamin A and COVID-19. SARS-CoV2 uses angiotensin-converting enzyme 2 (ACE2) for receptor-mediated cell entry, leading to ACE2 down-regulation and a deregulation of the renin–angiotensin system. Viral RNA triggers the production of CD4^+^ T-helper type 1 cells and cytokines, including TNF-*α*, IL-1, IL-6 and IL-18. In the lung, SARS-CoV2 can lead to severe acute respiratory syndrome due to extensive pulmonary fibrosis promoted by enhanced lipofibroblast–myofibroblast transition. Since lipofibroblasts rely on retinoids to initiate, coordinate and regulate alveolar septal eruption and alveolo-genesis, the loss of retinoids during the virus-induced lipofibroblast–myofibroblast transition could impair the ability of the lung to repair damaged epithelial surfaces, potentially leading to long-lasting scarring, lung fibrosis and reduced pulmonary capacity, which could manifest into a ‘long COVID’ effect. Treatment of COVID-19 patients with dexamethasone could further increase localised vitamin A deficiency through the reduction of retinoid-binding proteins and receptors. On the other hand, SARS-CoV2 could also lead to systemic vitamin A deficiency through a combination of increased urinary losses, reduced intake and absorption and increased utilisation. The effects of local and systemic vitamin A deficiency have been shown to reduce the ability of the immune system to maintain healthy respiratory and intestinal epithelial barriers, activity and numbers of type 1 innate lymphoid cells and secretory IgA responses to virus infection. This vicious cycle of increased vitamin A deficiency and decreased regulatory and protective immunity impairs recovery and is likely to increase morbidity and mortality.

VA is absorbed in the intestine with high efficiency in healthy individuals (>80 %) and mainly stored in the liver (>90 %), with smaller amounts stored in intestine, kidney, adipose tissue and the lung^(79)^. Release of serum VA bound to retinol-binding protein (RBP4) is homoeostatically controlled and does not change over a wide range of liver reserves^(79)^. During infection, the acute protein response increases the synthesis of hepatic inflammatory cytokines, but at the same time reduces the release of negative acute phase proteins such as RBP4. The subsequent reduction of holo-RBP4 (retinol bound to apo-RBP4) causes a decline in circulating VA even before the acute-phase proteins CRP and AGP have reached peak concentrations^(80)^. Reduced serum retinol and RBP4 concentrations could potentially overestimate the prevalence of VA deficiency in populations with high levels of inflammation^(81)^. Severe infections have also shown to reduce food intake^(80)^, impair absorption by 20–30 %^(15)^, increase metabolic requirements or catabolic losses^(80)^ and result in substantial urinary losses^(17,82)^. Importantly, respiratory infections combined with low dietary VA intake could deplete liver VA stores in COVID-19 patients suffering from pneumonia to levels associated with VA deficiency, since urinary losses of >1000 μg retinol/d have been observed in 24 % of ICU patients with pneumonia and sepsis, representing a higher amount than the RDA for VA^(82)^. We estimate that this level of urinary loss, combined with a postulated decrease in intake of approximately 50 % of the RDA for women hospitalised with COVID-19, could lead to a loss of approximately 1350 μg/d from liver stores. Such losses would lead to deficient liver stores (<20 μg/g) in approximately 3 weeks for a 61 kg women with relatively low pre-existing liver stores (40 μg/g; estimated liver weight of 2·4 % of body weight). Using data on estimated liver stores among US women of 129 (sd 89) μg/g who consumed high levels of VA (a mean intake of 173 (sd 111) % of the RDA of 700 μg retinol)^(83)^, we estimate that approximately 16 % of women (those at least one sd below the mean) in a population with similarly high intake would have reserves of 40 μg/g or less. In populations consuming lower levels of VA, the percentage of individuals at risk for systemic VA deficiency would be higher. It is important to note however that these systemic effects of VA are different to tissue-specific VA deficiency that could develop following COVID-19 infection (Fig. 1). First, pulmonary fibrosis induced by SARS-CoV2 is characterised by the conversion of lipofibroblasts to activated myofibroblasts that excessively deposit extracellular matrix proteins and lose their retinoid stores^(45,48)^. Second, inflammation induced reduction of retinoic acid uptake and metabolism to polar metabolites^(80)^ could affect the ability of the lung to regenerate from pulmonary fibrosis, since lipofibroblasts rely on retinoids to initiate, coordinate and regulate alveolar septal eruption and alveolo-genesis^(45)^. Third, the ability to deliver retinoids to the lung is compromised during severe infection due to the decline in circulating VA^(80)^. Nutritionists have long recognised that acute infection exacerbates the risk of malnutrition, as there is potential to fall into a ‘vicious circle’ of acute illness, deficiency and susceptibility to secondary infection. However, the timing of VA supplementation during SARS-CoV2 infection and the recovery period may be critical, as early intervention during the infectious period may be contraindicative due to its impact on the ACE2 receptor^(37,38)^, but intervention during the recovery period may be critical to address the potentially developing tissue-specific VA deficiency and its effects on the respiratory immune response. Importantly, VA supplementation may not be beneficial depending on age and co-morbidity, since VA supplementation can increase the risk of acute respiratory tract infections in children with adequate VA status^(84)^ and can delay recovery from community-acquired pneumonia in children^(85)^ and respiratory syncytial virus infection in infants^(86)^. However, VA supplementation was associated with improved recovery from Ebola virus infection in adults^(87)^ and can have beneficial effects in obesity by correcting tissue VA deficiency, altering lung immune cell composition, improving vaccine responses and assisting in respiratory virus clearance^(88)^. In particular, high-dose VA supplementation during recovery from measles has been shown to decrease mortality and improve recovery, including decreasing the severity of secondary bacterial pneumonia^(89)^. Delivering VA through inhalation may be more efficient to address the immunological lesions in VA-deficient individuals^(90,91)^ particularly since inflammation also reduces retinoic acid uptake and metabolism to polar metabolites^(80)^.

## Interaction of vitamin A with olfactory function

SARS-CoV2 causes olfactory and/or gustatory dysfunctions in COVID-19 patients^(92–95)^. A recent meta-analysis of eight studies confirmed that patients with COVID-19 had significantly higher risks of developing olfactory and/or gustatory dysfunctions compared with normal subjects (OR of 65·9) or patients with acute respiratory infection without detectable virus (OR of 11·3)^(94)^. On average, 50 % of COVID-19 patients suffered from olfactory and/or gustatory dysfunctions^(94)^, although olfactory impairment was reported to vary between 4·8 % in China, 68 % in the USA and 85·6 % in Europe^(93)^. The mean time from olfactory loss to recovery onset was reported as 12 d, with complete olfactory recovery in only 51 % of patients^(93)^. Viral infections are a common cause of olfactory dysfunction^(96)^, although the pathogenesis of sensory loss after viral infections is not well characterised^(92,95)^. It is assumed that the nasal epithelium and olfactory bulb could be critical in the pathological development of COVID-19, with mild outcomes of COVID-19 leading to localised effects with mild effects on olfactory function. Interestingly, the effects of SARS-CoV2 on chronic olfactory impairment increase with age, affecting up to 50 % of people aged ≥65 years and >80 % of people aged ≥80 years^(92)^.

VA supplementation may play an important role in the regeneration of olfactory receptor neurons, particularly since retinoic acid signalling is involved in neuronal regeneration^(97)^. Indeed, a recent retrospective cohort analysis in patients with post-infectious olfactory dysfunction indicated that topically applied VA at a dose of 10 000 IU/d for 8 weeks improved clinical outcome^(98)^. This beneficial effect of VA to improve olfactory dysfunction is supposedly due to increased local concentration of VA at the olfactory neuroepithelium^(98)^. Thus, similarly for treating VA deficiency in the lung, topical administration of VA may increase bioavailability and reduce toxic side effects of high-dose systemic VA therapy needed to overcome localised VA deficiency^(91,98,99)^. However, since no studies have investigated the benefit of topical application of VA in COVID-19 patients suffering from olfactory dysfunction, prospective, double-blind and placebo-controlled trials are needed to confirm if VA therapy would be of benefit to those patients that struggle to recover from olfactory impairment.

## Interaction of vitamin A with current SARS-CoV2 medication

The RECOVERY trial results demonstrated that low-dose dexamethasone reduced the death rate of patients that require respiratory support^(3)^. However, although corticosteroids have anti-inflammatory, antioxidant, pulmonary vasodilator and anti-edematous effects^(100)^, their impact on limiting immune responses might counteract viral clearance^(101)^. Previous studies associate the use of corticosteroid in SARS, MERS and influenza with a higher risk of a delayed viral clearance and long-term complications in survivors^(102)^. Furthermore, dexamethasone decreases alveolar numbers, and this effect on alveolar architecture can be long term or permanent^(18)^. The drug also reduces retinoid-binding proteins and receptors in mice^(18)^, which potentially could affect the metabolism of VA in affected tissues. Since chronic nutritional VA deficiency can result in pulmonary fibrosis^(14)^, long-term complications in survivors after dexamethasone treatment could include lung tissue scaring due to medication-induced VA deficiency. On the other hand, supplementation with a nutrient-metabolite combination containing VA and retinoic acid in a 10:1 molar ratio (VARA) was effective in increasing the concentration of VA to a level that was not antagonised by dexamethasone, probably since the VARA-induced lung tissue VA accumulation compensated the VA loss caused by dexamethasone^(103)^. Importantly, the delivery of retinoids to the lung tissue determines whether or not the negative side effects of dexamethasone on lung VA levels can be overcome. Treatment with retinoic acid was shown to reverse pulmonary emphysema^(104)^, but caused significant toxic side effects^(105)^. To obtain biologically effective levels in the lung, inhalation of retinoids rather than oral administration may be a better approach^(91)^, particularly since this approach induces cellular retinol-binding protein 1 protein expression which was shown to be reduced following dexamethasone treatment^(18)^. However, effectiveness of either VARA or retinoic acid inhalation to optimise lung VA stores would need to be tested in COVID-19-affected individuals who have been treated with dexamethasone.

## Summary

Traditionally, VA plays a very important role in the response of the immune system to infection, and this role may be particularly important in the development of a protective immune response after a SARS-CoV2 infection has occurred. This stresses the need for an adequate VA status prior to infection in order to support the immune system in its ability to deal with the inflammatory response due to SARS-CoV2 via a range of mechanisms that have been summarised in this review. However, in spite of an adequate *a priori* VA reserve, SARS-CoV2 infection itself may diminish VA stores. Such an infection-induced VA deficiency may impair the ability of the lung to repair damaged epithelial surfaces, potentially leading to long-lasting scarring, lung fibrosis and reduced pulmonary capacity. Thus, VA supplementation during recovery may be needed even though VA supplementation early in infection may not have been indicated due to a lack of VA deficiency prior to infection. In addition, earlier VA supplementation may have unpredictable effects for the following reasons: First, it is plausible that VA supplementation at the time of SARS-CoV2 exposure would increase the risk or severity of an infection since the cellular infection mechanism of SARS-CoV2 is mainly mediated by ACE2 which can be up-regulated by retinoic acid, as discussed above. Second, however, activation of ACE2 in infected patients could reduce the risk of sympathetic over-activation seen in severe SARS-CoV2 infection, and in particular in obese and diabetic COVID-19 patients. Topical application of VA may be beneficial to patients suffering from post-infectious olfactory dysfunction, since VA supplementation may play an important role in the regeneration of olfactory receptor neurons. Finally, there could be a beneficial use of VA as a co-medication in severe SARS-CoV2-affected patients who are treated with corticosteroids, particularly since dexamethasone was shown to negatively affect VA metabolism in lung tissues. It is plausible to assume that VA deficiency in at risk population groups would increase the risk of severe COVID-19 infection, but data to confirm this are currently missing. Importantly, COVID-19 has doubled the number of people in lower- and middle-income countries facing acute food insecurity and is predicted to reduce the coverage of VA supplementation programmes to VAD-affected populations between 15 and 50 %^(106)^. Since the risk of SARS-CoV2 infection is low in under 5-year-olds, and the benefit of VA supplementation outweighs the risk of developing a severe COVID-19 infection due to increased VA intake, VA supplementation programmes should continue for this age group.

## References

[1] Fung TS & Liu DX (2019) Human coronavirus: host–pathogen interaction. Annu Rev Microbiol 73, 529–557. 31226023 10.1146/annurev-micro-020518-115759

[2] Chen Y , Liu Q & Guo D (2020) Emerging coronaviruses: genome structure, replication, and pathogenesis. J Med Virol 92, 418–423. 31967327 10.1002/jmv.25681 PMC7167049

[3] Florindo HF , Kleiner R , Vaskovich-Koubi D , et al. (2020) Immune-mediated approaches against COVID-19. Nat Nanotechnol 15, 630–645. 32661375 10.1038/s41565-020-0732-3 PMC7355525

[4] WHO Coronavirus Disease (COVID-19) dashboard. https://covid19.who.int/ (accessed December 2020).

[5] Gandhi RT , Lynch JB & Del Rio C (2020) Mild or moderate Covid-19. N Engl J Med 383, 1757–1766. 32329974 10.1056/NEJMcp2009249

[6] Berlin DA , Gulick RM & Martinez FJ (2020) Severe Covid-19. N Engl J Med 383, 2451–2460. 32412710 10.1056/NEJMcp2009575

[7] Yadav R , Aggarwal S & Singh A (2020) SARS-CoV-2–host dynamics: increased risk of adverse outcomes of COVID-19 in obesity. Diabetes Metab Syndr 14, 1355–1360. 32755835 10.1016/j.dsx.2020.07.030 PMC7372253

[8] Johnson RM & Vinetz JM (2020) Dexamethasone in the management of Covid-19. BMJ 370, m2648. 32620554 10.1136/bmj.m2648

[9] Hu TY , Lee JZ & Asirvatham SJ (2020) Cardiovascular considerations in coronavirus disease 2019 with a special focus on arrhythmia. J Innovations Card Rhythm Manage 11, 4191–4198. 10.19102/icrm.2020.110804 PMC7452737 32874745

[10] Paniri A , Hosseini MM , Rasoulinejad A , et al. (2020) Molecular effects and retinopathy induced by hydroxychloroquine during SARS-CoV-2 therapy: role of CYP450 isoforms and epigenetic modulations. Eur J Pharmacol 886, 173454. 32763298 10.1016/j.ejphar.2020.173454 PMC7402235

[11] Green HN & Mellanby E (1928) Vitamin A as an anti-infective agent. Br Med J 2, 691–696. 20774205 10.1136/bmj.2.3537.691 PMC2456524

[12] Larange A & Cheroutre H (2016) Retinoic acid and retinoic acid receptors as pleiotropic modulators of the immune system. Annu Rev Immunol 34, 369–394. 27168242 10.1146/annurev-immunol-041015-055427

[13] Guo Y , Brown C , Ortiz C , et al. (2015) Leukocyte homing, fate, and function are controlled by retinoic acid. Physiol Rev 95, 125–148. 25540140 10.1152/physrev.00032.2013 PMC4281589

[14] Timoneda J , Rodríguez-Fernández L , Zaragozá R , et al. (2018) Vitamin A deficiency and the lung. Nutrients 10, 1132. 10.3390/nu10091132 PMC6164133 30134568

[15] Aklamati EK , Mulenga M , Dueker SR , et al. (2010) Accelerator mass spectrometry can be used to assess vitamin A metabolism quantitatively in boys in a community setting. J Nutr 140, 1588–1594. 20660280 10.3945/jn.110.125500 PMC3139233

[16] Gieng SH , Green MH , Green JB , et al. (2007) Model-based compartmental analysis indicates a reduced mobilization of hepatic vitamin A during inflammation in rats. J Lipid Res 48, 904–913. 17235116 10.1194/jlr.M600528-JLR200

[17] Stephensen CB (2001) Vitamin A, infection, and immune function. Annu Rev Nutr 21, 167–192. 11375434 10.1146/annurev.nutr.21.1.167

[18] Hind M & Maden M (2004) Retinoic acid induces alveolar regeneration in the adult mouse lung. Eur Respir J 23, 20–27. 14738226 10.1183/09031936.03.00119103

[19] Kuba K , Imai Y , Rao SA , et al. (2005) A crucial role of angiotensin converting enzyme 2 (ACE2) in SARS coronavirus-induced lung injury. Nat Med 11, 875–879. 16007097 10.1038/nm1267 PMC7095783

[20] Hoffmann M , Begon M , Lafon Y , et al. (2020) Influence of glenohumeral joint muscle insertion on moment arms using a finite element model. Comput Methods Biomech Biomed Engin 23, 1117–1126. 32643408 10.1080/10255842.2020.1789606

[21] Hikmet F , Mear L , Edvinsson A , et al. (2020) The protein expression profile of ACE2 in human tissues. Mol Syst Biol 16, e9610. 32715618 10.15252/msb.20209610 PMC7383091

[22] Nawijn MC & Timens W (2020) Can ACE2 expression explain SARS-CoV-2 infection of the respiratory epithelia in COVID-19? Mol Syst Biol 16, e9841. 32715628 10.15252/msb.20209841 PMC7383087

[23] Hamming I , Cooper ME , Haagmans BL , et al. (2007) The emerging role of ACE2 in physiology and disease. J Pathol 212, 1–11. 17464936 10.1002/path.2162 PMC7167724

[24] Gagliardi MC , Tieri P , Ortona E , et al. (2020) ACE2 expression and sex disparity in COVID-19. Cell Death Discovery 6, 37. 10.1038/s41420-020-0276-1 PMC7248455 32499922

[25] Kuba K , Imai Y & Penninger JM (2006) Angiotensin-converting enzyme 2 in lung diseases. Curr Opin Pharmacol 6, 271–276. 16581295 10.1016/j.coph.2006.03.001 PMC7106490

[26] Sodhi CP , Jenny N , Yamaguchi Y , et al. (2019) A dynamic variation of pulmonary ACE2 is required to modulate neutrophilic inflammation in response to pseudomonas aeruginosa lung infection in mice. J Immunol 203, 3000–3012. 31645418 10.4049/jimmunol.1900579 PMC7458157

[27] Tikellis C & Thomas MC (2012) Angiotensin-converting enzyme 2 (ACE2) is a key modulator of the renin angiotensin system in health and disease. Int J Pept 2012, 256294. 22536270 10.1155/2012/256294 PMC3321295

[28] Saheb Sharif-Askari N , Saheb Sharif-Askari F , Alabed M , et al. (2020) Airways expression of SARS-CoV-2 receptor, ACE2, and TMPRSS2 is lower in children than adults and increases with smoking and COPD. Mol Ther Methods Clin Dev 18, 1–6. 32537478 10.1016/j.omtm.2020.05.013 PMC7242205

[29] Ahmed F , Jones DB & Jackson AA (1990) The interaction of vitamin A deficiency and rotavirus infection in the mouse. Br J Nutr 63, 363–373. 2334670 10.1079/bjn19900122

[30] Paquette NC , Zhang LY , Ellis WA , et al. (1996) Vitamin A deficiency enhances ozone-induced lung injury. Am J Physiol 270, L475–L482. 8638741 10.1152/ajplung.1996.270.3.L475

[31] Stephensen CB , Blount SR , Schoeb TR , et al. (1993) Vitamin A deficiency impairs some aspects of the host response to influenza A virus infection in BALB/c mice. J Nutr 123, 823–833. 8487093 10.1093/jn/123.5.823

[32] Cone RA (2009) Barrier properties of mucus. Adv Drug Delivery Rev 61, 75–85. 10.1016/j.addr.2008.09.008 19135107

[33] Martin TR & Frevert CW (2005) Innate immunity in the lungs. Proc Am Thorac Soc 2, 403–411. 16322590 10.1513/pats.200508-090JS PMC2713330

[34] Richardson M (2003) The physiology of mucus and sputum production in the respiratory system. Nurs Times 99, 63–64. 12838653

[35] Koo JS , Jetten AM , Belloni P , et al. (1999) Role of retinoid receptors in the regulation of Mucin gene expression by retinoic acid in human tracheobronchial epithelial cells. Biochem J 338, 351–357. 10024510 PMC1220060

[36] Fan X , Liu S , Liu G , et al. (2015) Vitamin A deficiency impairs Mucin expression and suppresses the Mucosal immune function of the respiratory tract in chicks. PLOS ONE 10, e0139131. 26422233 10.1371/journal.pone.0139131 PMC4589363

[37] Zhong JC , Huang DY , Yang YM , et al. (2004) Upregulation of angiotensin-converting enzyme 2 by all-*trans* retinoic acid in spontaneously hypertensive rats. Hypertension 44, 907–912. 15477383 10.1161/01.HYP.0000146400.57221.74

[38] Zhou T-B , Wu W-F , Qin Y-H , et al. (2013) Association of all-*trans* retinoic acid treatment with the renin–angiotensin aldosterone system expression in glomerulosclerosis rats. J Renin-Angiotensin-Aldosterone Syst 14, 299–307. 23144044 10.1177/1470320312465220

[39] Fang L , Karakiulakis G & Roth M (2020) Are patients with hypertension and diabetes mellitus at increased risk for COVID-19 infection? Lancet Respir Med 8, E54–E54. 32171062 10.1016/S2213-2600(20)30116-8 PMC7118626

[40] Aksoy H , Karadag AS & Wollina U (2020) Angiotensin IIreceptors: impact for COVID-19 severity. Dermatol Ther 33, e13989. 32645228 10.1111/dth.13989 PMC7361069

[41] Mery G , Epaulard O , Borel A-L , et al. (2020) COVID-19: underlying adipokine storm and angiotensin 1–7 umbrella. Front Immunol 11, 1714. 32793244 10.3389/fimmu.2020.01714 PMC7385229

[42] Porzionato A , Emmi A , Barbon S , et al. (2020) Sympathetic activation: a potential link between comorbidities and COVID-19. FEBS J 287, 3681–3688. 32779891 10.1111/febs.15481 PMC7405290

[43] Marquez HA & Chen F (2020) Retinoic acid signaling and development of the respiratory system. Sub-Cell Biochem 95, 151–174. 10.1007/978-3-030-42282-0_6 32297299

[44] Wang S , Yu J , Kane MA , et al. (2020) Modulation of retinoid signaling: therapeutic opportunities in organ fibrosis and repair. Pharmacol Ther 205, 107415. 31629008 10.1016/j.pharmthera.2019.107415 PMC7299003

[45] Hind M , Gilthorpe A , Stinchcombe S , et al. (2009) Retinoid induction of alveolar regeneration: from mice to man? Thorax 64, 451–457. 19401491 10.1136/thx.2008.105437

[46] Tsuchida T & Friedman SL (2017) Mechanisms of hepatic stellate cell activation. Nat Rev Gastroenterol Hepatol 14, 397–411. 28487545 10.1038/nrgastro.2017.38

[47] Habiel DM & Hogaboam CM (2017) Heterogeneity of fibroblasts and myofibroblasts in pulmonary fibrosis. Curr Pathobiol Rep 5, 101–110. 29082111 10.1007/s40139-017-0134-x PMC5654579

[48] Kruglikov IL & Scherer PE (2020) The role of adipocytes and adipocyte-like cells in the severity of COVID-19 infections. Obesity 28, 1187–1190. 32339391 10.1002/oby.22856 PMC7267593

[49] Belvisi MG , Hele DJ & Birrell MA (2006) Peroxisome proliferator-activated receptor gamma agonists as therapy for chronic airway inflammation. Eur J Pharmacol 533, 101–109. 16458290 10.1016/j.ejphar.2005.12.048

[50] Sariol A & Perlman S (2020) Lessons for COVID-19 immunity from other coronavirus infections. Immunity 53, 248–263. 32717182 10.1016/j.immuni.2020.07.005 PMC7359787

[51] Hou YJ , Okuda K , Edwards CE , et al. (2020) SARS-CoV-2 reverse genetics reveals a variable infection gradient in the respiratory tract. Cell 182, 429–446.e414. 32526206 10.1016/j.cell.2020.05.042 PMC7250779

[52] Vabret N , Britton GJ , Gruber C , et al. (2020) Immunology of COVID-19: current state of the science. Immunity 52, 910–941. 32505227 10.1016/j.immuni.2020.05.002 PMC7200337

[53] Subbarao K & Mahanty S (2020) Respiratory virus infections: understanding COVID-19. Immunity 52, 905–909. 32497522 10.1016/j.immuni.2020.05.004 PMC7237932

[54] Ross AC & Stephensen CB (1996) Vitamin A and retinoids in antiviral responses. FASEB J 10, 979–985. 8801180

[55] Oliveira LM , Teixeira FME & Sato MN (2018) Impact of retinoic acid on immune cells and inflammatory diseases. Mediators Inflammation 2018, 3067126. 10.1155/2018/3067126 PMC6109577 30158832

[56] Vellozo NS , Pereira-Marques ST , Cabral-Piccin MP , et al. (2017) All-*trans* retinoic acid promotes an M1- to M2-phenotype shift and inhibits macrophage-mediated immunity to *Leishmania major* . Front Immunol 8, 1560. 29204144 10.3389/fimmu.2017.01560 PMC5698282

[57] Gundra UM , Girgis NM , Gonzalez MA , et al. (2017) Vitamin A mediates conversion of monocyte-derived macrophages into tissue-resident macrophages during alternative activation. Nat Immunol 18, 642–653. 28436955 10.1038/ni.3734 PMC5475284

[58] Xavier-Elsas P , Vieira BM , Masid-de-Brito D , et al. (2019) The need to consider context in the evaluation of anti-infectious and immunomodulatory effects of vitamin A and its derivatives. Curr Drug Targets 20, 871–878. 30556501 10.2174/1389450120666181217095323

[59] Murphy K , Weaver C (2016) Janeway’s Immunobiology, 9th ed New York: Garland Science/Taylor & Francis Group, LLC.

[60] Altmann DM & Boyton RJ (2020) SARS-CoV-2 T cell immunity: specificity, function, durability, and role in protection. Sci Immunol 5, eabd6160. 32680954 10.1126/sciimmunol.abd6160

[61] Yang L , Peng H , Zhu Z , et al. (2007) Persistent memory CD4^+^ and CD8^+^ T-cell responses in recovered severe acute respiratory syndrome (SARS) patients to SARS coronavirus M antigen. J Gen Virol 88, 2740–2748. 17872527 10.1099/vir.0.82839-0 PMC2362397

[62] Channappanavar R , Fett C , Zhao J , et al. (2014) Virus-specific memory CD8 T cells provide substantial protection from lethal severe acute respiratory syndrome coronavirus infection. J Virol 88, 11034–11044. 25056892 10.1128/JVI.01505-14 PMC4178831

[63] Zhao J , Alshukairi AN , Baharoon SA , et al. (2017) Recovery from the Middle East respiratory syndrome is associated with antibody and T-cell responses. Sci Immunol 2, eaan5393. 28778905 10.1126/sciimmunol.aan5393 PMC5576145

[64] Li CK , Wu H , Yan H , et al. (2008) T cell responses to whole SARS coronavirus in humans. J Immunol 181, 5490–5500. 18832706 10.4049/jimmunol.181.8.5490 PMC2683413

[65] Huang AT , Garcia-Carreras B , Hitchings MDT , et al. (2020) A systematic review of antibody mediated immunity to coronaviruses: kinetics, correlates of protection, and association with severity. Nat Commun 11, 4704. 32943637 10.1038/s41467-020-18450-4 PMC7499300

[66] Iwata M (2009) Retinoic acid production by intestinal dendritic cells and its role in T-cell trafficking. Semin Immunol 21, 8–13. 18849172 10.1016/j.smim.2008.09.002

[67] Stephensen CB , Moldoveanu Z & Gangopadhyay NN (1996) Vitamin A deficiency diminishes the salivary immunoglobulin A response and enhances the serum immunoglobulin G response to influenza A virus infection in BALB/c mice. J Nutr 126, 94–102. 8558330 10.1093/jn/126.1.94

[68] Penkert RR , Surman SL , Jones BG , et al. (2016) Vitamin A deficient mice exhibit increased viral antigens and enhanced cytokine/chemokine production in nasal tissues following respiratory virus infection despite the presence of FoxP3^+^ T cells. Int Immunol 28, 139–152. 26507129 10.1093/intimm/dxv064 PMC5892019

[69] Bakdash G , Vogelpoel LT , van Capel TM , et al. (2015) Retinoic acid primes human dendritic cells to induce gut-homing, IL-10-producing regulatory T cells. Mucosal Immunol 8, 265–278. 25027601 10.1038/mi.2014.64

[70] Schuster GU , Kenyon NJ & Stephensen CB (2008) Vitamin A deficiency decreases and high dietary vitamin A increases disease severity in the mouse model of asthma. J Immunol 180, 1834–1842. 18209081 10.4049/jimmunol.180.3.1834

[71] Brown CC , Esterhazy D , Sarde A , et al. (2015) Retinoic acid is essential for Th1 cell lineage stability and prevents transition to a Th17 cell program. Immunity 42, 499–511. 25769610 10.1016/j.immuni.2015.02.003 PMC4372260

[72] Liang Y , Yi P , Wang X , et al. (2020) Retinoic acid modulates hyperactive T cell responses and protects vitamin A-deficient mice against persistent lymphocytic choriomeningitis virus infection. J Immunol 204, 2984–2994. 32284332 10.4049/jimmunol.1901091 PMC7371257

[73] Seo GY , Jang YS , Kim HA , et al. (2013) Retinoic acid, acting as a highly specific IgA isotype switch factor, cooperates with TGF-beta1 to enhance the overall IgA response. J Leukocyte Biol 94, 325–335. 23744644 10.1189/jlb.0313128

[74] Mora JR & von Andrian UH (2009) Role of retinoic acid in the imprinting of gut-homing IgA-secreting cells. Semin Immunol 21, 28–35. 18804386 10.1016/j.smim.2008.08.002 PMC2663412

[75] Lo MW , Kemper C & Woodruff TM (2020) COVID-19: complement, coagulation, and collateral damage. J Immunol 205, 1488–1495. 32699160 10.4049/jimmunol.2000644 PMC7484432

[76] Lee JS , Park S , Jeong HW , et al. (2020) Immunophenotyping of COVID-19 and influenza highlights the role of type I interferons in development of severe COVID-19. Sci Immunol 5, eabd1554. 32651212 10.1126/sciimmunol.abd1554 PMC7402635

[77] Mangalmurti N & Hunter CA (2020) Cytokine storms: understanding COVID-19. Immunity 53, 19–25. 32610079 10.1016/j.immuni.2020.06.017 PMC7321048

[78] Catanzaro M , Fagiani F , Racchi M , et al. (2020) Immune response in COVID-19: addressing a pharmacological challenge by targeting pathways triggered by SARS-CoV-2. Signal Transduction Targeted Ther 5. 10.1038/s41392-020-0191-1 PMC7255975 32467561

[79] Olson JA (1990) Vitamin A. In *Present Knowledge in Nutrition*, 6th ed., pp. 96–107 [ML Brown, editor]. Washington, DC: International Life Sciences Institute-Nutrition Foundation.

[80] Rubin LP , Ross AC , Stephensen CB , et al. (2017) Metabolic effects of inflammation on vitamin A and carotenoids in humans and animal models. Adv Nutr 8, 197–212. 28298266 10.3945/an.116.014167 PMC5347109

[81] Larson LM , Guo J , Williams AM , et al. (2018) Approaches to assess vitamin A status in settings of inflammation: Biomarkers Reflecting Inflammation and Nutritional Determinants of Anemia (BRINDA) project. Nutrients 10, 1100. 10.3390/nu10081100 PMC6115742 30115830

[82] Stephensen CB , Alvarez JO , Kohatsu J , et al. (1994) Vitamin A is excreted in the urine during acute infection. Am J Clin Nutr 60, 388–392. 8074070 10.1093/ajcn/60.3.388

[83] Valentine AR , Davis CR & Tanumihardjo SA (2013) Vitamin A isotope dilution predicts liver stores in line with long-term vitamin A intake above the current recommended dietary allowance for young adult women. Am J Clin Nutr 98, 1192–1199. 24047915 10.3945/ajcn.113.063867 PMC3798076

[84] Grotto I , Mimouni M , Gdalevich M , et al. (2003) Vitamin A supplementation and childhood morbidity from diarrhea and respiratory infections: a meta-analysis. J Pediatr 142, 297–304. 12640379 10.1067/mpd.2003.116

[85] Stephensen CB , Franchi LM , Hernandez H , et al. (1998) Adverse effects of high-dose vitamin A supplements in children hospitalized with pneumonia. Pediatrics 101, E3. 10.1542/peds.101.5.e3 9565436

[86] Bresee JS , Fischer M , Dowell SF , et al. (1996) Vitamin A therapy for children with respiratory syncytial virus infection: a multicenter trial in the United States. Pediatr Infect Dis J 15, 777–782. 8878220 10.1097/00006454-199609000-00008

[87] Aluisio AR , Perera SM , Yam D , et al. (2019) Vitamin A supplementation was associated with reduced mortality in patients with Ebola virus disease during the West African outbreak. J Nutr 149, 1757–1765. 31268140 10.1093/jn/nxz142 PMC6768816

[88] Penkert RR , Cortez V , Karlsson EA , et al. (2020) Vitamin A corrects tissue deficits in diet-induced obese mice and reduces influenza infection after vaccination and challenge. Obesity (Silver Spring) 28, 1631–1636. 32779401 10.1002/oby.22929 PMC7483416

[89] Hussey GD & Klein M (1990) A randomized, controlled trial of vitamin-A in children with severe measles. N Engl J Med 323, 160–164. 2194128 10.1056/NEJM199007193230304

[90] McGill JL , Kelly SM , Guerra-Maupome M , et al. (2019) Vitamin A deficiency impairs the immune response to intranasal vaccination and RSV infection in neonatal calves. Sci Rep 9, 15157. 31641172 10.1038/s41598-019-51684-x PMC6805856

[91] Schaeffer MW , Roy SS , Mukherjee S , et al. (2010) Uptake of all-*trans* retinoic acid-containing aerosol by inhalation to lungs in a guinea pig model system – a pilot study. Exp Lung Res 36, 593–601. 21043991 10.3109/01902141003790155 PMC3058486

[92] Gori A , Leone F , Loffredo L , et al. (2020) COVID-19-related anosmia: the olfactory pathway hypothesis and early intervention. Front Neurol 11, 956. 33013637 10.3389/fneur.2020.00956 PMC7511833

[93] Gorzkowski V , Bevilacqua S , Charmillon A , et al. (2020) Evolution of olfactory disorders in COVID-19 patients. Laryngoscope 130, 2667–2673. 32617990 10.1002/lary.28957 PMC7361712

[94] Hoang MP , Kanjanaumporn J , Aeumjaturapat S , et al. (2020) Olfactory and gustatory dysfunctions in COVID-19 patients: a systematic review and meta-analysis. Asian Pac J Allergy Immunol 38, 162–169. 32563232 10.12932/AP-210520-0853

[95] Lechien JR , Chiesa-Estomba CM , De Siati DR , et al. (2020) Olfactory and gustatory dysfunctions as a clinical presentation of mild-to-moderate forms of the coronavirus disease (COVID-19): a multicenter European study. Eur Arch Oto-Rhino-Laryngol 277, 2251–2261. 10.1007/s00405-020-05965-1 PMC7134551 32253535

[96] Murphy C , Doty RL & Duncan HJ (2003) Clinical disorders of olfaction In Handbook of Olfaction and Gustation, 3rd ed, pp. 461–478 [ RL Doty , editor]. New York: Marcel Dekker.

[97] Rawson NE & LaMantia AS (2007) A speculative essay on retinoic acid regulation of neural stem cells in the developing and aging olfactory system. Exp Gerontol 42, 46–53. 16860961 10.1016/j.exger.2006.05.021

[98] Hummel T , Whitcroft KL , Rueter G , et al. (2017) Intranasal vitamin A is beneficial in post-infectious olfactory loss. Eur Arch Oto-Rhino-Laryngol 274, 2819–2825. 10.1007/s00405-017-4576-x 28434127

[99] Brooks AD , Tong W , Benedetti F , et al. (2000) Inhaled aerosolization of all-*trans*-retinoic acid for targeted pulmonary delivery. Cancer Chemother Pharmacol 46, 313–318. 11052629 10.1007/s002800000148

[100] Mokra D , Mikolka P , Kosutova P , et al. (2019) Corticosteroids in acute lung injury: the dilemma continues. Int J Mol Sci 20, 4765. 10.3390/ijms20194765 PMC6801694 31557974

[101] Lai ST (2005) Treatment of severe acute respiratory syndrome. Eur J Clin Microbiol Infect Dis 24, 583–591. 16172857 10.1007/s10096-005-0004-z PMC7088345

[102] Huang Q , Wu X , Zheng X , et al. (2020) Targeting inflammation and cytokine storm in COVID-19. Pharmacolog Res 159, 105051. 10.1016/j.phrs.2020.105051 PMC7320704 32603772

[103] Ross AC & Ambalavanan N (2007) Retinoic acid combined with vitamin A synergizes to increase retinyl ester storage in the lungs of newborn and dexamethasone-treated neonatal rats. Neonatology 92, 26–32. 17596734 10.1159/000100083 PMC3843127

[104] Massaro GD & Massaro D (1997) Retinoic acid treatment abrogates elastase-induced pulmonary emphysema in rats. Nat Med 3, 675–677. 9176496 10.1038/nm0697-675

[105] Lippman SM , Lee JJ , Karp DD , et al. (2001) Randomized phase III intergroup trial of isotretinoin to prevent second primary tumors in stage I non-small-cell lung cancer. JNCI-J Nat Cancer Inst 93, 605–618. 10.1093/jnci/93.8.605 11309437

[106] Headey D , Heidkamp R , Osendarp S , et al. (2020) Impacts of COVID-19 on childhood malnutrition and nutrition-related mortality. Lancet 396, 519–521. 32730743 10.1016/S0140-6736(20)31647-0 PMC7384798

